# The Impact of Breast Implant Cohesivity on Rippling and Revision Procedures in 2-Stage Prepectoral Breast Reconstruction

**DOI:** 10.1093/asjof/ojae028

**Published:** 2024-04-23

**Authors:** Neil Parikh, Goutam K Gadiraju, Matthew Prospero, Yizhuo Shen, Bryce F Starr, Erik Reiche, Colby J Hyland, Sarah J Karinja, Justin M Broyles

## Abstract

**Background:**

Rippling remains one of the most common complications following prepectoral implant-based reconstruction (IBR).

**Objectives:**

The purpose of this study was to assess how implant cohesivity, a measure of elasticity and form stability, affects the incidence of rippling in prepectoral IBR.

**Methods:**

We performed a retrospective cohort study of 2-stage prepectoral IBR performed between January 2020 and June 2022 at the Brigham and Women's Hospital and Dana-Farber Cancer Institute, comparing outcomes in patients who received Allergan Natrelle least cohesive, moderately cohesive, and most cohesive silicone gel implants. Outcomes of interest were rippling and reoperation for fat grafting.

**Results:**

A total of 129 patients were identified, of whom 52 had the least cohesive implants, 24 had the moderately cohesive implants, and 53 patients had the most cohesive implants. The mean follow-up time was 463 (±220) days. A decreased incidence of rippling was seen with moderately cohesive (odds ratio [OR] 0.30, *P* < .05) and most cohesive (OR 0.39, *P* < .05) implants. Third stage reoperation for fat grafting was less frequent in patients with the most cohesive implant (OR 0.07, *P* < .05). In subgroup analyses, the patients with the most cohesive implant, who did not receive fat grafting at the time of initial implant placement, did not require reoperation for fat grafting (0%).

**Conclusions:**

The use of highly cohesive implants in prepectoral IBR is associated with decreased rippling and fewer reoperations for fat grafting.

**Level of Evidence: 3:**

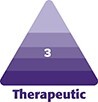

In the United States, implant-based reconstruction (IBR) is one of the most widely used methods for breast reconstruction and the management of postmastectomy breast defects. Traditionally, this reconstruction is performed in a 2-stage fashion, with the first stage involving tissue expanders.^1^ The 2 common approaches to 2-stage IBR are subpectoral and prepectoral implant placement. The subpectoral approach involves placing the implant under the pectoralis muscle without releasing the inferior origin of the muscle.^2^ The prepectoral approach involves placement of the implant above the pectoralis major muscle, within a subcutaneous pocket.^3^ Although the subpectoral approach has historically been the most common, recent studies have demonstrated that the prepectoral approach is a safe and reliable alternative.^4,5^ For example, it has been noted that the early complications and implant explantation rates are comparable between these 2 reconstructive approaches.^3^

Prepectoral implant-based breast reconstruction continues to gain traction among both patients and surgeons.^6^ In a recent survey of the American Society of Plastic Surgeons, 75.70% of respondents reported having performed prepectoral reconstruction in their practice.^7^ Prepectoral prosthetic placement in an appropriate candidate can reduce postoperative pain, eliminate animation deformity, and mitigate functional discomfort in comparison with subpectoral reconstruction.^8,9^ These benefits, however, are at times counterbalanced by findings of an increased incidence of postoperative rippling deformity, which are often associated with mastectomy type, smaller implant size, and thinner skin flaps.^10,11^ We hypothesize that implant cohesivity may also play a critical role.

Cohesivity, an attribute of the silicone gel found within breast implants, refers to the form stability of the gel, or the ability of an implant to maintain its shape and dimensions regardless of position.^12^ On a molecular level, cohesivity corresponds to the degree of crosslinking between the silicone polymers that comprise the gel. A less cohesive gel is less crosslinked and more elastic; a more cohesive gel is more crosslinked and less elastic. This is clinically relevant as more cohesive implants are thought to provide improved superior pole contour in the upright position, primarily because of diminished redistribution of the gel to the dependent inferior portion of the implant or breast pocket; thereby, affording greater control over volume distribution to produce an aesthetic shape.^13^ There is limited data regarding the association between cohesivity and rippling in prepectoral IBR.

Significant rippling can be problematic and may decrease patient satisfaction.^14^ If rippling is severe enough, it may warrant surgical correction with implant exchange, pocket conversion, and/or autologous fat grafting.^15,16^ The purpose of this study is to compare the incidence of rippling and reoperation with autologous fat grafting as they relate to implant cohesivity. We hypothesize that the increased form stability of highly cohesive implants will minimize rippling at the superior pole in prepectoral breast reconstruction and result in fewer reoperations for autologous fat grafting.

## METHODS

### Study Design

A retrospective cohort study of females who had undergone 2-stage IBR following mastectomy (unilateral or bilateral, skin or nipple-sparing) was conducted to determine the effect of implant cohesivity on postoperative rippling and reoperation for fat grafting. This study followed the Strengthening the Reporting of Observational Studies in Epidemiology guidelines.^17^ The study was approved by the Institutional Review Board of Mass General Brigham (IRB Protocol 2022P001507).

### Setting and Participants

An institutional breast oncology database was used to identify females who had undergone 2-stage IBR and were treated at the Brigham and Women's Hospital and Dana-Farber Cancer Institute between January 2020 and June 2022. Patients were included if they were females 18 years and older and had undergone 2-stage prepectoral IBR. Females with follow-up duration less than 6 months post-IBR at the time of data collection were excluded to allow for sufficient time for observing postoperative complications and the need for revision surgery. The electronic medical record was reviewed to obtain the demographic and clinical data for the study. Participants were classified into 3 study cohorts based on implant cohesivity: Allergan Natrelle Responsive (ie, TruForm 1 or least cohesive), SoftTouch (ie, TruForm 2 or moderately cohesive), and Cohesive (ie, TruForm 3 or most cohesive) (Allergan, Santa Barbara, CA). The least cohesive implant group was used as the reference or control cohort.

### Outcomes

The primary study outcomes were rippling and the incidence of third stage revision reoperation for autologous fat grafting. The presence of rippling was determined through a chart review of follow-up visit documentation. Patients were recorded as having developed the outcome if their plastic surgeon explicitly used the word “rippling” in documenting physical examination findings. Third stage revision-reoperation for autologous fat grafting was identified by reviewing patients' surgical histories. Specifically, this outcome referred to autologous fat grafting performed as a revision procedure following implant placement. Postoperative complications included infection and capsular contracture. Both outcomes were captured by reviewing all available follow-up visit documentation for the explicit mention of either complication.

### Statistical Methods

The primary and secondary outcomes of interest for each cohort were collected, and statistical analysis was performed using R programming software version 4.2.2 (The R Foundation, Vienna, Austria). Specifically, continuous variables were analyzed using analysis of variance. Categorical data were analyzed using a 2-tailed Fisher's exact test. Univariate regression models were performed to calculate odds ratios (ORs) with CIs. Statistical significance was assigned to values of *P* < .05.

## RESULTS

### Participants

A total of 235 females met the inclusion criteria. Of these patients, 106 were excluded given inadequate follow-up duration. Thus, a total of 129 patients were included in the analysis and represented patients from a heterogeneous group of 8 surgical oncologists and 8 plastic surgeons. All patients received Natrelle silicone breast implants (Allergan, Santa Barbara, CA). Fifty-two (40%) patients received Responsive (ie, TruForm 1 or least cohesive) implants, 24 (19%) patients received SoftTouch (ie, TruForm 2 or moderately cohesive) implants, and 53 (41%) patients received Cohesive (ie, TruForm 3 or most cohesive) implants. The mean age of the patients was 48.5 (±10.5) years. The mean BMI was 25.31 (±4.94). Patients were followed for a mean of 463 (±220) days after the second stage, permanent implant placement. Further patient demographic and clinical characteristics are shown in Table 1.

**Table 1.** Patient Demographics and Clinical Characteristics

Characteristic			
Implant type	Responsive	SoftTouch	Cohesive
No. of patients	52	24	53
Mean age, in years (range, SD)	48 (27-68, 11)	47 (33-73, 9)	49 (31-68, 10)
Mean BMI (range, SD)	25.33 (16.45-38.00, 4.86)	25.78 (18.80-38.40, 6.17)	25.08 (17.70-39.30, 4.40)
Mean follow-up, in days (range, SD)	468 (187-1130, 230)	417 (186-901, 183)	478 (181-930, 227)
Race, *n* (%)			
White	46 (88.5)	22 (91.7)	46 (86.8)
Black	1 (1.9)	0 (0.0)	3 (5.7)
Asian	1 (1.9)	0 (0.0)	3 (5.7)
Other	1 (1.9)	2 (8.3)	1 (1.9)
Unknown	3 (5.8)	0 (0.0)	0 (0.0)
Ethnicity, *n* (%)			
Hispanic	0 (0.0)	1 (4.2)	0 (0.0)
Non-Hispanic	51 (98.1)	22 (91.7)	50 (94.3)
Unknown	1 (1.9)	1 (4.2)	3 (5.7)
Hypertension, *n* (%)	14 (26.9)	3 (12.5)	4 (7.5)
Diabetes, *n* (%)	2 (3.8)	0 (0.0)	3 (5.7)
Smoker, *n* (%)			
Active	0 (0.0)	0 (0.0)	3 (5.7)
Former	14 (26.9)	2 (8.3)	14 (26.4)
Never	38 (73.1)	22 (91.7)	35 (66.0)
Unknown	0 (0.0)	0 (0.0)	1 (1.9)
Mean implant size, in cc (range, SD)	449 (140-770, 147)	413 (145-775, 164)	410 (175-770, 136)
Acellular dermal matrix placement, *n* (%)	51 (98.1)	24 (100)	53 (100)
Rippling following implant placement, *n* (%)	21 (40.4)	4 (16.7)	11 (20.8)
Fat grafting after implant placement, *n* (%)	11 (21.2)	2 (8.3)	1 (1.9)
Rippling resolved with fat grafting, *n* (%)	4 (7.7)	0 (0.0)	0 (0.0)
Infection after tissue expander placement, *n* (%)	3 (5.8)	0 (0.0)	1 (1.9)
Infection after breast implant placement, *n* (%)	4 (7.7)	0 (0.0)	1 (1.9)
Capsular contracture, *n* (%)	4 (7.7)	0 (0.0)	0 (0.0)

### Outcomes

Across all 3 cohorts, 36 patients (28%) experienced rippling after implant placement, 72 patients (56%) received fat grafting at the time of tissue expander removal and implant placement, and 14 patients (11%) received fat grafting at a third stage reoperation after implant placement. Rippling resolved in 4 of the patients who received fat grafting at a third stage reoperation. With respect to complications, across all 3 cohorts, 4 patients (3%) experienced an infection after tissue expander placement, 5 patients (4%) experienced an infection after breast implant placement, and 4 patients (3%) developed capsular contracture. There was no significant correlation between age and BMI with regards to implant cohesivity.

Irrespective of whether patients underwent fat grafting at the time of implant placement, those who received the moderately cohesive (OR 0.30, *P* < .05) and most cohesive (OR 0.39, *P* < .05) implants were less likely to experience rippling compared with the patients who received the least cohesive implant (Table 2). Patients, who received the most cohesive implant, irrespective of whether they received fat grafting at the time of implant placement, were less likely to require additional sessions of fat grafting after implant placement when compared with those who received the moderately cohesive or least cohesive implant options (OR 0.07, *P* < .05; Table 3).

**Table 2.** The Patients who Received the Moderately Cohesive and Most Cohesive Implants were Significantly Less Likely to Experience Rippling Complications Compared to Those who Received the Least Cohesive Implants

Characteristic	OR^a^	95% CI	*P*-value
Implant type			
Responsive	—	—	
SoftTouch	0.30	0.08-0.92	.048
Cohesive	0.39	0.16-0.90	.031

^a^OR, odds ratio.

**Table 3.** The Patients who Received the Most Cohesive Implants were Significantly Less Likely to Require Additional Fat Grafting Compared to Those who Received the Moderately Cohesive and Least Cohesive Implants

Characteristic	OR^a^	95% CI	*P*-value
Implant type			
Responsive	—	—	
SoftTouch	0.34	0.05-1.41	.183
Cohesive	0.07	0.00-0.39	.013

^a^OR, odds ratio.

A subgroup analysis was performed, studying the 57 patients who did not receive fat grafting at the time of second stage implant placement. Within this patient cohort, 12 patients received the most cohesive implant, 10 patients received the moderately cohesive implant, and 35 patients received the least cohesive implant. Zero of the 12 patients (0%) from the most cohesive cohort underwent third stage fat grafting. In contrast, patients who received the least cohesive implant were significantly more likely to undergo third stage fat grafting (11 of the 35 patients [31%]; *P* < .05, Table 4).

**Table 4.** The Patients who Received the Moderately Cohesive and Most Cohesive Implants, Without Fat Grafting at the Time of the Implant Placement, were Significantly Less Likely to Require Additional Fat Grafting Compared to Those who Received the Least Cohesive Implants

	Implant type, *n* (%)			Total	*P*-value^a^
	Responsive	SoftTouch	Cohesive		
Additional fat grafting					.032
No	24 (42)	9 (16)	12 (21)	45 (79)	
Yes	11 (19)	1 (1.8)	0 (0)	12 (21)	
Total	35 (61)	10 (18)	12 (21)	57 (100)	

^a^Fisher's exact test.

## DISCUSSION

The purpose of this study was to investigate the impact of breast implant cohesivity on the incidence of rippling in prepectoral IBR and the need for reoperation for fat grafting. We found that the use of highly cohesive implants is associated with decreased rippling and fewer reoperations for fat grafting. This may potentially translate into cost savings in providing higher value care, whereby incremental increases in implant costs may offset the need for additional procedures. We believe that these findings will have a significant impact on the future clinical decision making and considerations of physicians and their patients.

Rippling is defined as either a visible or palpable cutaneous manifestation of a breast implant, in which the contour is evident through the skin, and may appear as patterns of irregularities or undulations.^16^ Vidya et al created a novel grading system for rippling in implant-based complications. Grade 1 rippling, the lowest grade, was defined as no evidence of rippling seen both at rest and with movement, whereas Grade 4 rippling, the highest grade, was defined as severe-persistent rippling causing gross deformity both at rest and with movement. In applying this system to a clinical setting, they found that rippling was most often associated with low BMI and poor subcutaneous fat preoperatively.^18^ Alongside advancements in the evaluation of rippling, there exist a variety of techniques to correct this complication. Examples of existing treatment options, include implant upsizing, fat grafting, the use of acellular dermal matrix, and maintaining a small pocket.^15,16^ However, a relatively unexplored area of rippling management is the variation in implant cohesivity.

Generally, modern-day silicone implants are cohesive in the sense that the silicone filler is viscous. The viscosity or cohesivity of the filler impacts the distribution of the silicone gel within the implant, thereby influencing the shape and dimension.^19^ A retrospective chart review by Brown et al evaluated 32 patients who underwent breast reconstruction with cohesive gel implants. They found that cohesive gel implants have the potential to minimize the risk of postoperative rippling, create a more natural breast shape, and provide a greater degree of safety in case of loss of implant integrity.^20^ In 2007, Panettiere et al performed a prospective clinical study to compare soft cohesive prostheses vs lower cohesivity silicone prostheses in the context of breast augmentation. They found that 9.2% of patients in the soft cohesive group experienced rippling, whereas 55.0% of the patients in the lower cohesive group experienced rippling.^21^ Our study builds on this previous literature by presenting the largest patient cohort used to analyze the impact of implant cohesivity on rippling, specifically in prepectoral breast reconstruction. We believe that our findings will motivate clinicians and researchers to consider and explore the impact of such unique breast implant characteristics when educating and advising patients.

Our study has strong clinical translatability because of a variety of factors. First, our data included a heterogeneous patient population that was followed for an appropriate timeframe of at least 6 months in order to capture the complications and outcomes in question. Furthermore, the inclusion of patients treated by several surgeons at a single institution amplifies the translatability of our results. The implants included in the study are from a single manufacturer, which helps to eliminate several potential implant product confounders. As surgeons often choose to use implants with the same cohesivity in a majority of their cases, our study aims to elucidate the benefit of further evaluating implant cohesivity as a variable that may improve patients' outcomes after prepectoral IBR.

Our study is not without its limitations, including those inherent in retrospective chart review. Rippling is often inversely associated with breast flap thickness in prepectoral patients, which unfortunately was not possible to control retrospectively. Individual surgeon and patient preferences regarding implant cohesivity and the time of autologous fat grafting might affect our results and cannot be adjusted for. Lastly, our group captured the incidence of rippling through a patient chart review. Therefore, patients who might have suffered rippling complications, but lacked documentation in their charts, might have been excluded from analysis. A prospective study that employs randomized implant assignment and a standardized protocol for fat grafting in order to evaluate the impact of cohesivity will address many of these limitations.

## CONCLUSIONS

Prepectoral IBR affords preservation of the chest wall and minimizes animation deformity when compared with traditional techniques but is frequently complicated by rippling because of the interaction of the implant with overlying soft tissues. By analyzing the largest retrospective patient cohort to date used to assess the impact of implant cohesivity on rippling following prepectoral IBR, this study demonstrates that the use of highly cohesive implants is significantly associated with fewer complications of rippling and revisional procedures. These data suggest that highly cohesive implants may confer a greater value to patients undergoing prepectoral IBR by reducing the need for revisional procedures.
